# Modeling of wear resistance for TC21 Ti-alloy using response surface methodology

**DOI:** 10.1038/s41598-023-31699-1

**Published:** 2023-03-21

**Authors:** Ali Abdelmoneim, Ramadan N. Elshaer, M. El-Shennawy, Arafa S. Sobh

**Affiliations:** 1 Faculty of Engineering, Helwan University, Cairo, Egypt; 2 Tabbin Institute for Metallurgical Studies, Cairo, Egypt

**Keywords:** Engineering, Materials science, Mathematics and computing

## Abstract

This study investigated the effect of heat treatment processes on the dry sliding wear resistance of the TC21 Ti-alloy at several levels of normal load and sliding speed. Response Surface Methodology (RSM) has been used as a design of the experiment procedure. OM and FESEM besides XRD analysis were used for results justification. Highest hardness of 49 HRC was recorded for WQ + Aging specimens due to the plenty of *α*″ which decomposed to α_s_ and the more α_s_, while the lowest hardness of 36 HRC was reported for WQ specimens. The results revealed that specimens subjected to water quenching and aging (WQ + Aging) under extreme load and speed conditions (50 N and 3 m/s), possessed the poorest wear resistance although they had the highest hardness. While those left in the annealed condition revealed the highest wear resistance although they had much lower hardness when compared to other conditions. A mathematical polynomial model for wear resistance expressed in wear rate was developed, validated then used to get the optimum parameters.

## Introduction

Several engineering applications require engineers to get materials with high strength, stiffness, fracture toughness, and extreme service temperatures with low weight^1^. This collection of properties can easily be supported by titanium (Ti) and its alloys. As a result, their range of applications is extended to include advanced engineering applications in construction, automotive, power generation, biomedical, chemical processing, aerospace, and marine industries^2,3^. However, titanium and its alloys encounter difficulties when used in field of wear and friction. This is attributed to their low wear resistance and high chemical affinity under certain circumstances compared to steels^4^. TC21 is a newly developed damage-tolerant Ti alloy with high specific strength and service temperature^5^. It belongs to α + β alloys that represent more than 70% of the Ti alloys market^6^. This is because these alloys can be strengthened by thermal and thermomechanical treatments. Hence, a wide range of microstructures and mechanical properties can be obtained to customize applications^7^. TC21 is thought to be a strong competitor and replacement for the well-known Ti-alloy Ti–6Al–4V (Ti64)^8^. Some call Ti64 a workhorse titanium industry alloy, it dominates 50% of the global market^6^. Although both alloys are α + β alloys, but TC21 has a higher specific strength and fracture toughness than Ti64 alloy. The application filed for TC21 involves aerospace products such as landing gear components, load-bearing structures, engine shafts, fuselages, and frames^9^.

The wear behavior of TC21 has been investigated from both sliding wear and fretting wear perspectives. Elshear et al.^10^ investigated the effect of cooling rate and aging process on wear behavior of deformed TC21 Ti-alloy. The better combination of properties was achieved by air-cooled and aged (AC + aging) condition. In another work^11^, the authors studied the effect of cold deformation in addition to heat treatment. X. Guo et al.^4^ investigated the influence of single, double, and triple heat treatments on the microstructure and dry sliding wear properties of TC21 alloy. They found that wear resistance of α + β basket-weave microstructure (resulting from double and triple treatments) is higher than that of single-phase *β* microstructure. For fretting wear, results of Lin et al.^12^ revealed that the amplitude had the greatest influence on wear resistance when compared to both frequency and normal load. the damage mechanism was mainly abrasive wear mechanism. According to Yan et al.^13^, the fretting wear was conducted at elevated temperature (150 °C). The authors reported that the effect of temperature on the friction coefficient was displacement dependent. In addition, compared to room temperature, the wear rate was reduced by 67.4–86.5% and oxidation wear mechanism was the main mechanism. Far from using traditional heat treatment processes to control wear characteristics of the TC21 alloy, a lot of research^14–16^ reported the exploiting of surface modification technology and oxidation process to improve the hardness and wear resistance of the TC21 alloy.

In order to get valid, reliable conclusions along with keeping costs and time of experimental runs as minimum as possible Design of Experiments (DOE) is used extensively in tribological field as wear test is classified as a destructive test. One of the most used designs in either industry or research work is Taguchi designs or Taguchi orthogonal arrays, which can be used in both the process design and product stage to enhance manufacturability and reliability of the product^17^. Using Taguchi’s L9 orthogonal array design R. Sahoo et al.^1,18^ studied the influence of factors such as microstructural variation resulting from heat treatment process, sliding velocity, normal load, and test duration on dry sliding wear behavior of Ti–6Al–4V titanium alloy at room temperature. Paramjit Singh et al.^19^ also used Taguchi design with L25 orthogonal array to optimize the deep cryogenic treatment conditions for dry sliding behavior of the same alloy. Control factors included soaking durations (t_cs_), tempering temperatures (T_tp_), sliding speed (v_s_), contact pressure (p_c_), and sliding time (t_s_) with 5 levels for each one. Although Taguchi method reduces the total number of runs effectively, it doesn’t indicate precisely to the cause of variability in the response which could be due to main effects, interactions between control factors, or curvature^20^. Taguchi focuses on the main effects and gives less interest to interaction effects unless they are pre-assigned in the orthogonal array.

Several researchers utilized RSM as DOE technique to investigate wear behavior of Ti alloys. El-Tayeb et al.^21,22^ compared between frictional behavior of two α + β titanium alloys, Ti54 and Ti64, under both dry air and dry cryogenic (liquid N_2_) sliding conditions They used RSM to develop models describing the interrelation between the output responses-coefficient of friction and Wear volume-and the input variables. Chauhan et al.^23^ tried to emphasize the mechanisms responsible for the low wear resistance of the titanium (Grade 5) alloy. They used RSM to investigate the effect of three dry sliding factors on the specific wear rate and a predictive model has been developed. M. D. Sharma et al.^24^ modeled and optimized dry sliding friction and wear characteristics e.g., wear rate, average friction coefficient, and maximum contact temperature of Ti–3Al–2.5V alloy. The models were transformed, such as log or inverse square root of the response as a function of input variables. Babu et al.^25^ also, developed a reduced quadratic model to correlate specific wear rate of Ti–3Al–2.5V alloy under dry sliding conditions to some input variables such as load, speed and sliding distance. Elshaer et al.^26^ used RSM to analyze how pressure and velocity influenced the Abbott Firestone zones and wear behavior of low-carbon steel.

Literature reporting on using traditional heat treatment processes to control dry sliding wear behavior of the newly developed TC21 alloy is limited. This could be attributed to two reasons, the first one, Ti64 alloy is still the preferred Ti-alloy. The second reason, most of research related to wear behavior of TC21 alloy focuses on surface modification techniques although they have a lot of disadvantages. Those disadvantages include large expenses, complicated procedures, high energy consumption, and environmental hazards^27^. In addition, it was noticed that all researchers^4,10–13^, only investigate the effect of one input factor at a time on the wear characteristics. So, the current work aims to narrow this gap and to the best of authors’ knowledge, this is the first attempt to develop a regression model for the wear rate under dry sliding wear condition of TC21 Ti-alloy against high-speed steel (HSS) using RSM. In addition, the developed model after validation can be used to make predictions within the design space for optimization purposes which have been accomplished in our work.

## Experimental work

### Material and testing

The alloy under investigation is TC21 Ti-alloy supplied by *Baoji Hanz Material Technology Co., Ltd., China* with chemical composition shown in Table 1. With a diameter of 7 mm and a length of 140 mm, the alloy used in this study was annealed in rod form. β transus temperature of this alloy is 950 ± 5 °C^11^.

**Table 1:** Average chemical composition of studied TC21 Ti-alloy, wt. %.

Al	Mo	Zr	Sn	Nb	Cr	Si	Fe	Ti
6.09	2.94	2.12	2.06	1.96	1.46	0.09	0.06	Balance

**Figure 1:**
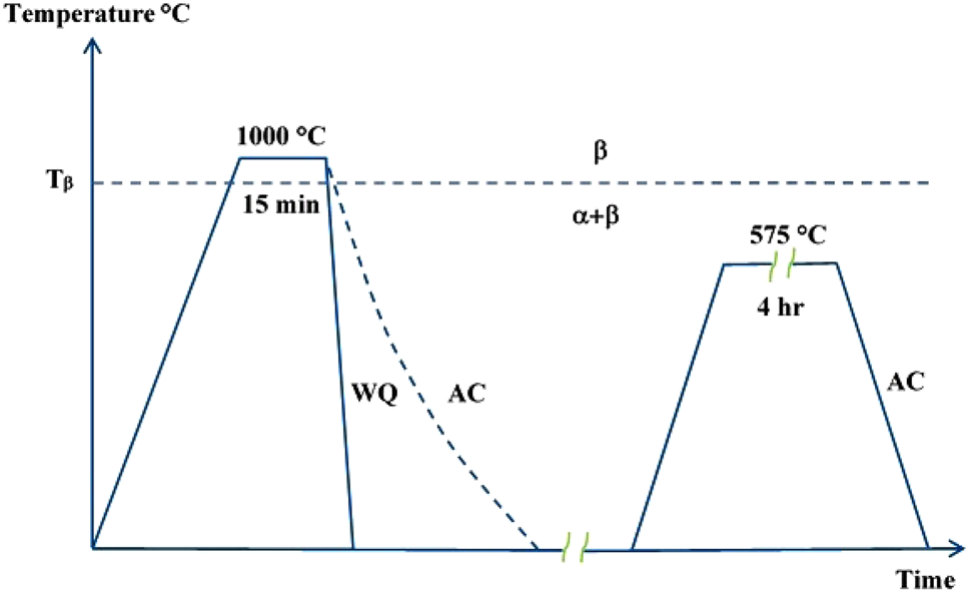
Cycles of different heat treatments.

There are 4 different heat treatment cycles used in this work, see Fig. 1. Table 2 summarizes the details of the heat treatment cycles. An electric programmable furnace (Muffle furnace/model HTC03/1) with a controlled atmosphere was used for all heat treatment cycles. To get specimens suitable for next different tests, the TC21 rods were cut into small specimens of 7 mm dimeter and 12 mm length by means of electrical discharge machining (EDM) wire cut machine (NOVICUT 350M model 2015). These small specimens were ground up to 1000 grit. For metallography examination purposes, specimen from every group were selected and embedded in cold mounting resin, ground, polished, and finally etched using 3% HF, 30% HNO_3_ and 67% H_2_O etchant composition. Optical Microscope (OM) was then used for metallurgical examination.

**Table 2:** Heat treatment processes details.

Cycle	Heat treatment cycles
1	1000 °C/15 min + AC, for short (AC)
2	1000 °C/15 min + WQ, for short (WQ)
3	1000 °C/15 min + AC + Aging (575 °C/4 h), for short (AC + Aging)
4	1000 °C/15 min + WQ + (Aging 575 °C/4 h), for short (WQ + Aging)

Rockwell hardness (scale C) test was carried out using Rockwell hardness tester (United True-Blue II model U-2004) according to ASTM E18 standards. Seven readings have been recorded for each specimen. Dry sliding wear test for 15 min at ambient temperature was carried out using pin on disk tester for selected specimens based on a design of experiments procedure. Wear specimens (Φ7 and 12 length) were fixed against high-speed steel (HSS) disk with hardness of 64 HRC. Before each individual run, the disk was ground with emery paper of 1000 grit, both the disk and specimen were cleaned with acetone, and then an air blower was used for drying and blowing any contaminations. To get the mass loss due to wear, an electronic balance with a resolution of 0.0001 g was used to weigh the specimen before and after the test. The wear resistance expressed by the wear rate (WR) is given by:

1 $$WR=\frac{\Delta m}{t}\quad {\text{g/min}}$$

where, Δm: mass loss in grams (g), t: time in minutes (min).

The test was repeated three times at the same levels of normal load and sliding speed then the average was determined and recorded. At the beginning of each run, each specimen was left for period of time until the surface is fully upset to the desk surface to get a uniform wear rate and avoid the effect of run-in period.

To identify and assess the wear mechanisms, Field emission scanning electron microscopy (FESEM) of worn-out surfaces was performed for some specimens under conditions of (10 N; 1.5 m/s) and (50 N; 3 m/s) which represent low and severe wear conditions, respectively. Also, some collected debris was optically examined.

### Design of experiments (DOE)

The output response that is interesting in this investigation is the wear resistance of the TC21, expressed in wear rate (WR). RSM is used to model the WR as a function of input parameters. According to Refs.^1,18,19,21–25^ there are many parameters that could influence the wear characteristics, such as normal load/pressure, sliding speed, sliding time/distance, material of the friction pairs, temperature, surface roughness, humidity, and lubrication. Among all of that, the load and sliding speed are the most influential ones.

**Figure 2:**
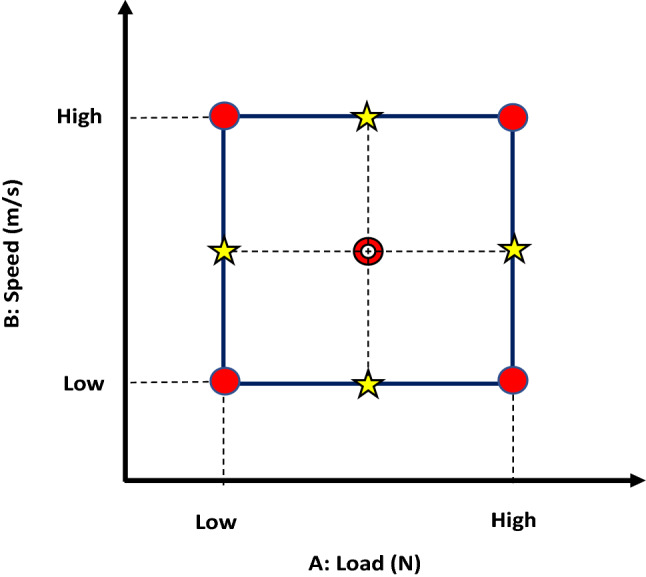
Face-centered central composite design.

The low and high levels of the input factors had been assigned based on the literature survey, considering the technical capabilities of the wear testing machine available. Table 3 illustrates the levels of the input factors. In this study, the face-centered central composite design (CCD), Fig. 2, was used to construct the design matrix. The face-centered CCD consists of a total of 11 points, detailed as 4 factorial, 4 axial, and 3 center points. These 11 points were used for each level of the categorical factor (heat treatment). So, we get 55 runs in total in the design matrix (Table 4). The Design Expert 13 software was used for purposes of DOE and subsequent statistical analysis.

**Table 3:** Input factors and levels.

Factor	Type	Code	Level				
			–	Low (− 1)	Medium (0)	High (+ 1)	–
Normal load (N)	Numeric	A	–	10	30	50	–
Sliding speed (m/s)	Numeric	B	–	1.5	2.25	3	–
Heat treatment	Categoric	C	Annealed	AC	WQ	AC + Aging	WQ + Aging

**Table 4:** Design matrix including input factors and corresponding responses.

Run	Factor 1 A: Load (N)	Factor 2 B: Speed (m/s)	Factor 3 C: Heat treatment	Response WR (g/min)	Run	Factor 1 A: Load (N)	Factor 2 B: Speed (m/s)	Factor 3 C: Heat treatment	Response WR (g/min)
1	10	1.5	Annealed	1.89	29	30	1.5	WQ	3.17
2	50	1.5		8.05	30	30	3		6.27
3	10	3		2.62	31	30	2.25		4.63
4	50	3		10.92	32	30	2.25		4.81
5	10	2.25		2.31	33	30	2.25		4.49
6	50	2.25		10.27	34	10	1.5	AC + Aging	2.07
7	30	1.5		3.54	35	50	1.5		8.77
8	30	3		5.46	36	10	3		2.5
9	30	2.25		4.81	37	50	3		29.72
10	30	2.25		5.33	38	10	2.25		2.32
11	30	2.25		5.22	39	50	2.25		17.82
12	10	1.5	AC	2.08	40	30	1.5		3.43
13	50	1.5		7.76	41	30	3		6.42
14	10	3		2.16	42	30	2.25		5.7
15	50	3		37.26	43	30	2.25		5.81
16	10	2.25		2.14	44	30	2.25		5.98
17	50	2.25		13.77	45	10	1.5	WQ + Aging	1.91
18	30	1.5		3.87	46	50	1.5		9.86
19	30	3		5.72	47	10	3		2.31
20	30	2.25		5.11	48	50	3		58.43
21	30	2.25		5.09	49	10	2.25		2.47
22	30	2.25		5.39	50	50	2.25		42.68
23	10	1.5	WQ	1.77	51	30	1.5		3.67
24	50	1.5		6.79	52	30	3		5.37
25	10	3		2.47	53	30	2.25		6.41
26	50	3		23.25	54	30	2.25		6.02
27	10	2.25		2.03	55	30	2.25		6.34
28	50	2.25		10.28					

## Results and discussion

### Microstructure evaluation

**Figure 3:**
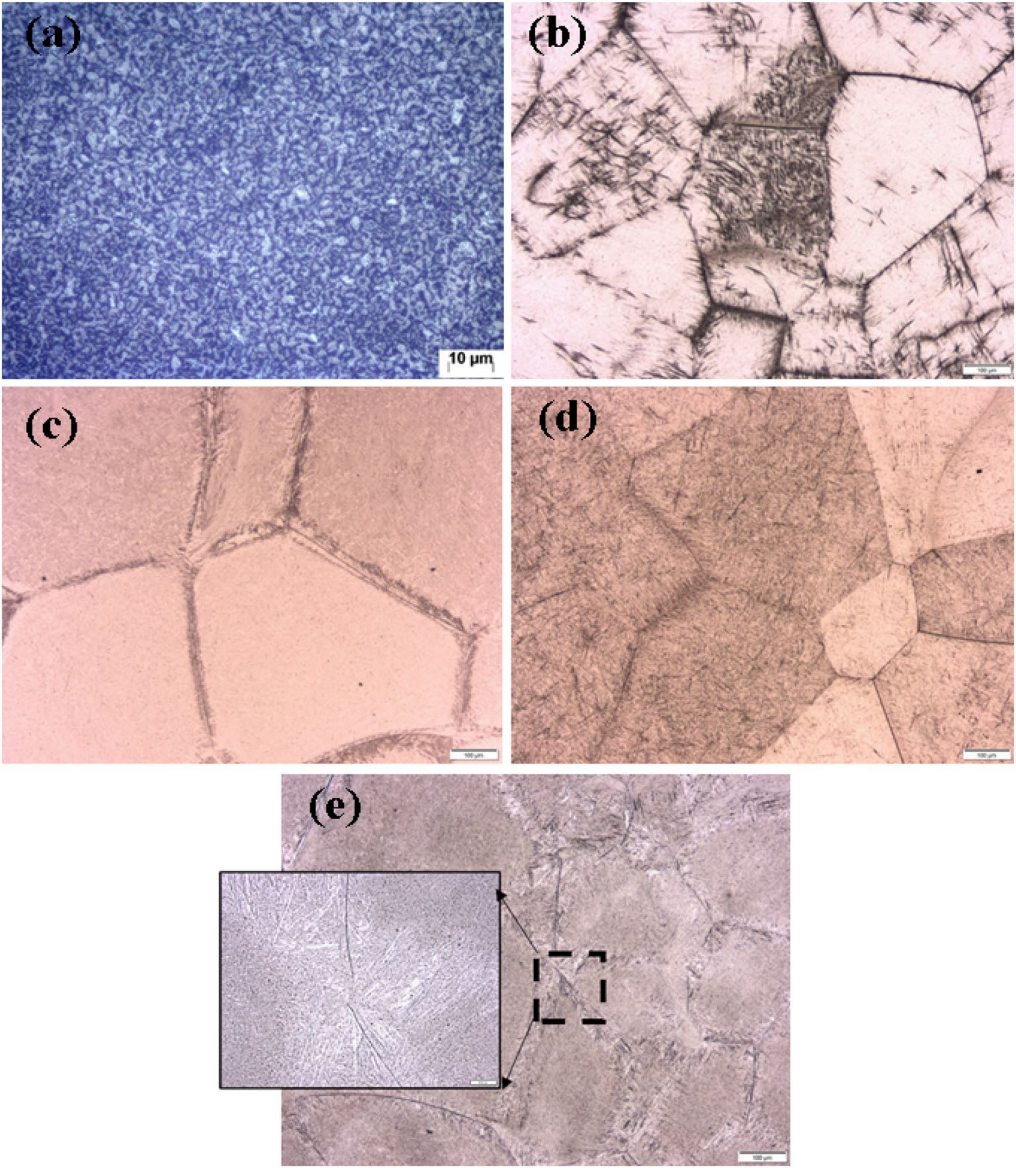
OM images of microstructure: (**a**) Annealed, (**b**) AC, (**c**) WQ, (**d**) AC + Aging and (**e**) WQ + Aging.

Figure 3 shows the microstructure of annealed and different heat treatment conditions. The microstructure of the annealed consists of equiaxed *α*-phase that is uniformly distributed within a matrix of *β*-phase (Fig. 3a). According to phase volume fraction analysis based on image processing, *α*-phase which is soft phase^1,28^, represents about 65% of volume and as a result, the annealed specimens were softer than treated ones except WQ. By heating above *β* transus temperature the whole *α* turned into *β*. If the alloy is AC to room temperature, coarse *α* plates form within *β* grains, Fig. 3b. While in case of rapid cooling i.e., WQ orthorhombic martensite (*α*″) forms, Fig. 3c^29^. Although it looks intuitive, the obvious decrease in the hardness of WQ samples is attributed to orthorhombic martensite *α*″, which contrary to *hcp* martensite *α*′, has a softening effect as previously reported^30^. By subjecting the cooled specimens to subsequent aging process, the coarse plates in AC specimens become finer) and some precipitations of secondary α (*α*_*s*_) form, Fig. 3d. While in WQ + Aging specimens, the *α*″ was totally decomposed to fine* α*_*s*_ and *β*^31,32^. These *α*_*s*_ dispersed within the *β* grain and become clearer along with the grain boundary, Fig. 3e.

### Hardness variations

**Figure 4:**
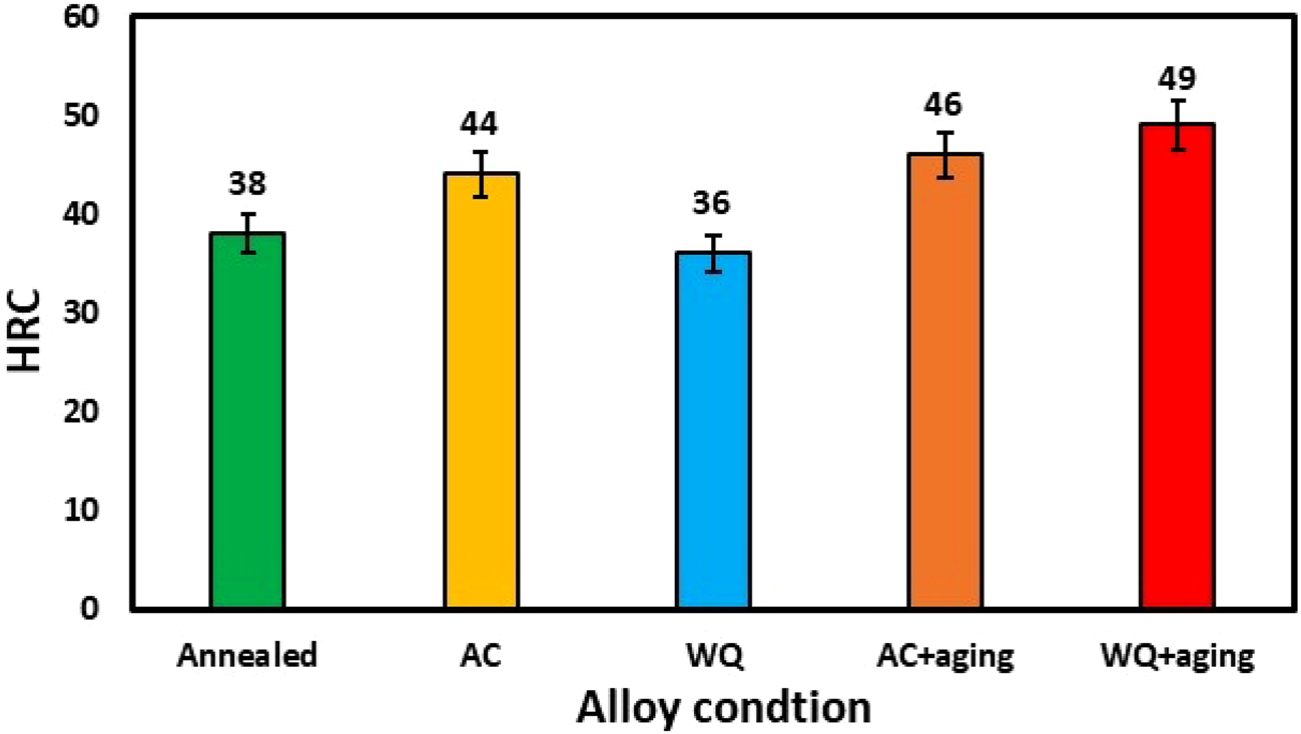
Hardness of TC21-Ti alloy at different conditions.

The different heat treatment processes resulted in a variety of microstructures. This induced a remarkable variation in the hardness of the treated specimens as illustrated in Fig. 4. Annealed specimens showed a hardness value of 38 HRC. WQ specimens revealed the lowest hardness value of 36 HRC. While WQ + Aging specimens obtained the high hardness of 49 HRC. This reflects about 36% increase in hardness as compared to WQ and WQ + Aging specimens. Therefore, specimens after WQ + Aging had the highest hardness due to the plenty of *α*″ which decomposed to α_s_ and the more α_s_, the more interphase boundaries, the more barriers to dislocation motion.

### Tribological analysis

#### Wear rates (WR)

**Figure 5:**
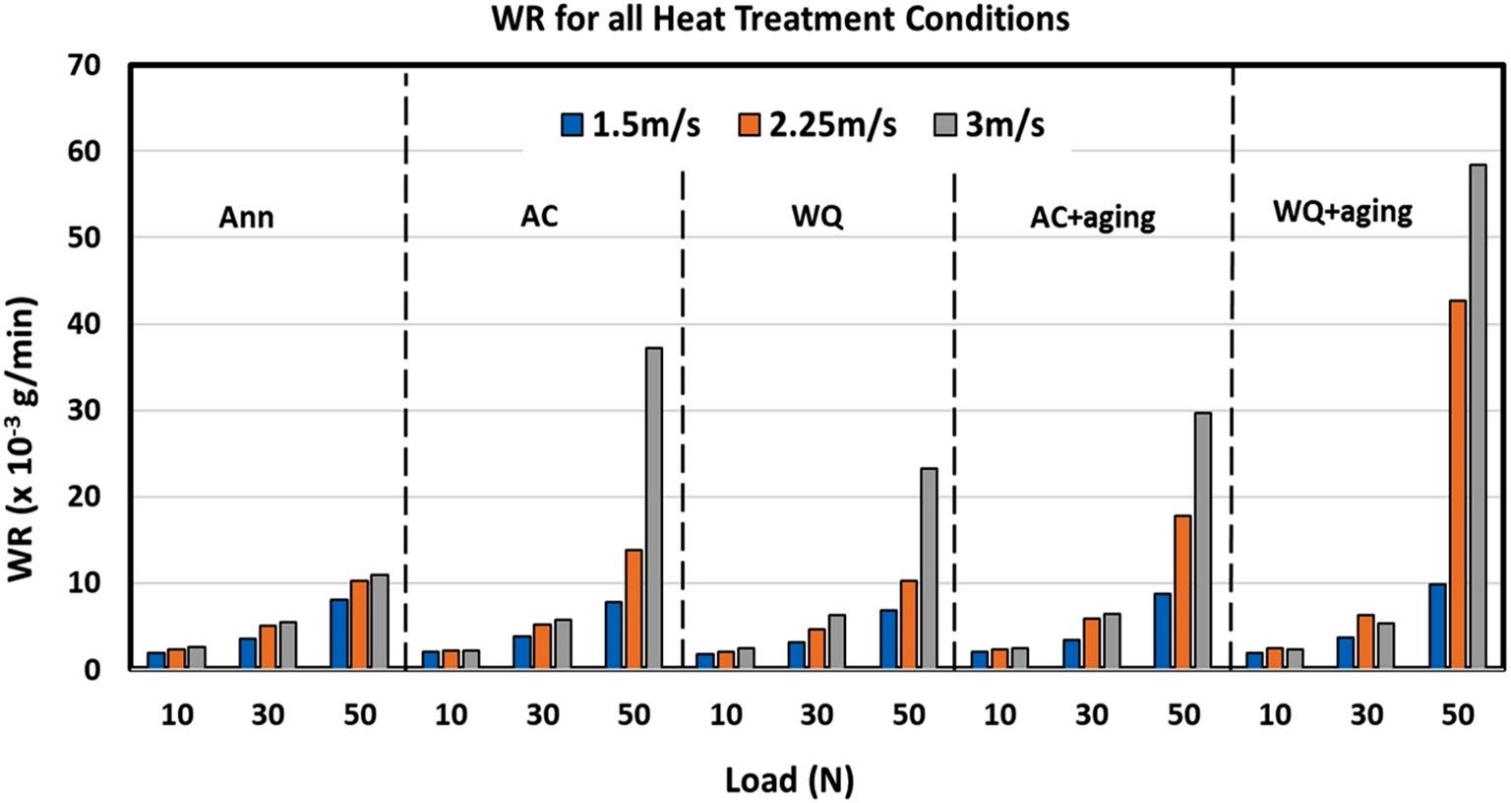
Wear rate for different conditions.

Figure 5 illustrates the wear rate for all treatment conditions of the TC21 Ti-alloy under all tested levels of both the normal load and sliding speed. One can conclude that the effect of sliding speed is limited for all treatment conditions under low and medium normal loads of 10 and 30 N, respectively. While under same speeds the effect of normal load was significant. For WQ + Aging specimens under 10 and 30 N, WR was increased then decreased when the speed increased from 1.5 to 2.25 m/s and then increased from 2.25 to 3 m/s, respectively. This may be attributed to the occurrence of adhesive wear which decreases by increasing the sliding speed which in turn reduces the time and opportunity of material diffusion between the two friction mates especially when no lubricant film is used. On the other hand, under severe normal load of 50 N, all treatment conditions showed a dramatic increase in WR when speed was increased from 1.5 to 3 m/s. This increase was as minimum as possible for the Annealed condition and as maximum as possible for WQ + Aging treatment.

Although the hardness of the annealed is much lower than that of WQ + Aging, the wear resistance of annealed samples is higher than that of WQ + Aging under the same combination of high load and speed. This seems to be intuitive specially when compared to competitive materials such as steels, but the microstructure variation resulting from different heat treatments and the frictional thermal effect occurring upon these extreme conditions of testing play an important role in this unfamiliar behavior.

**Figure 6:**
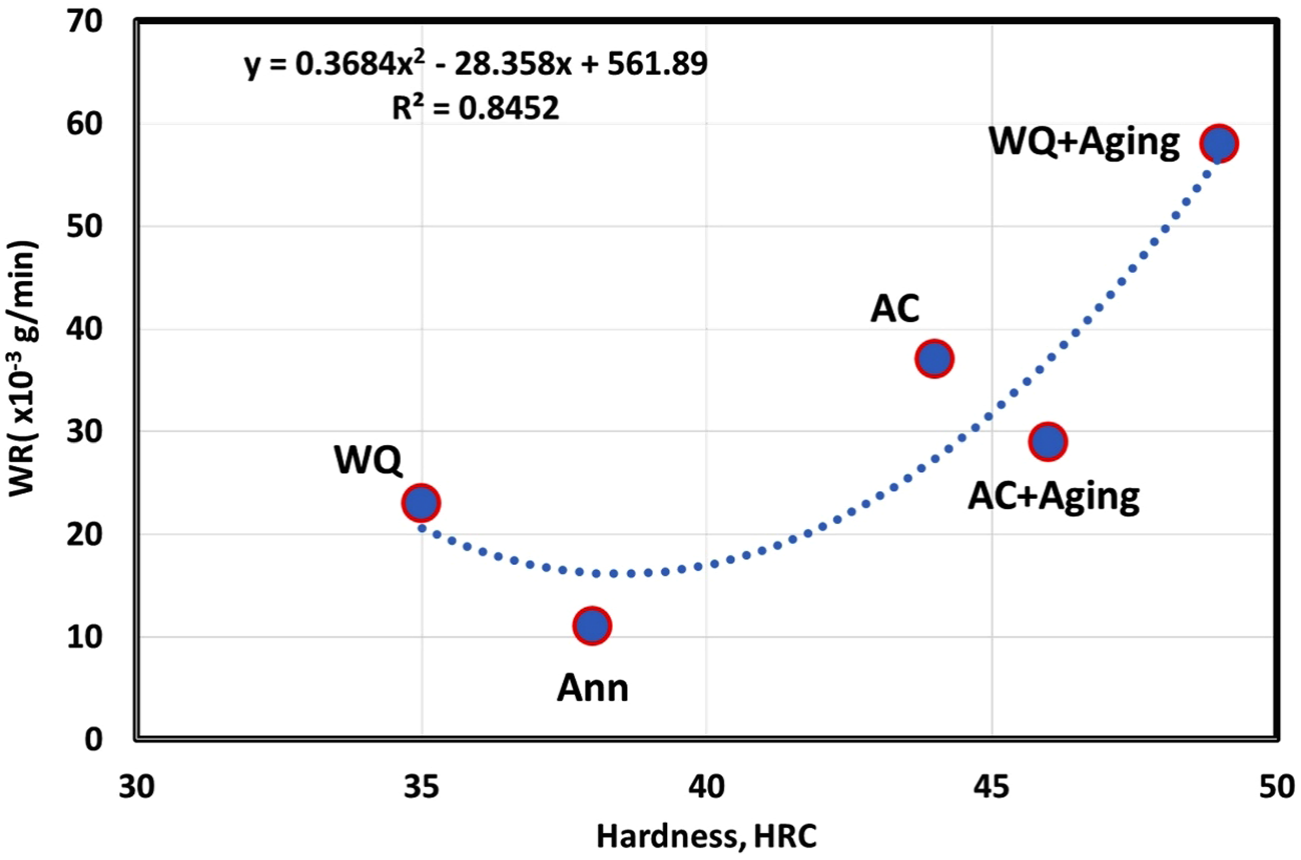
Correlation between hardness and Wear rate at extreme conditions of load and speed.

**Figure 7:**
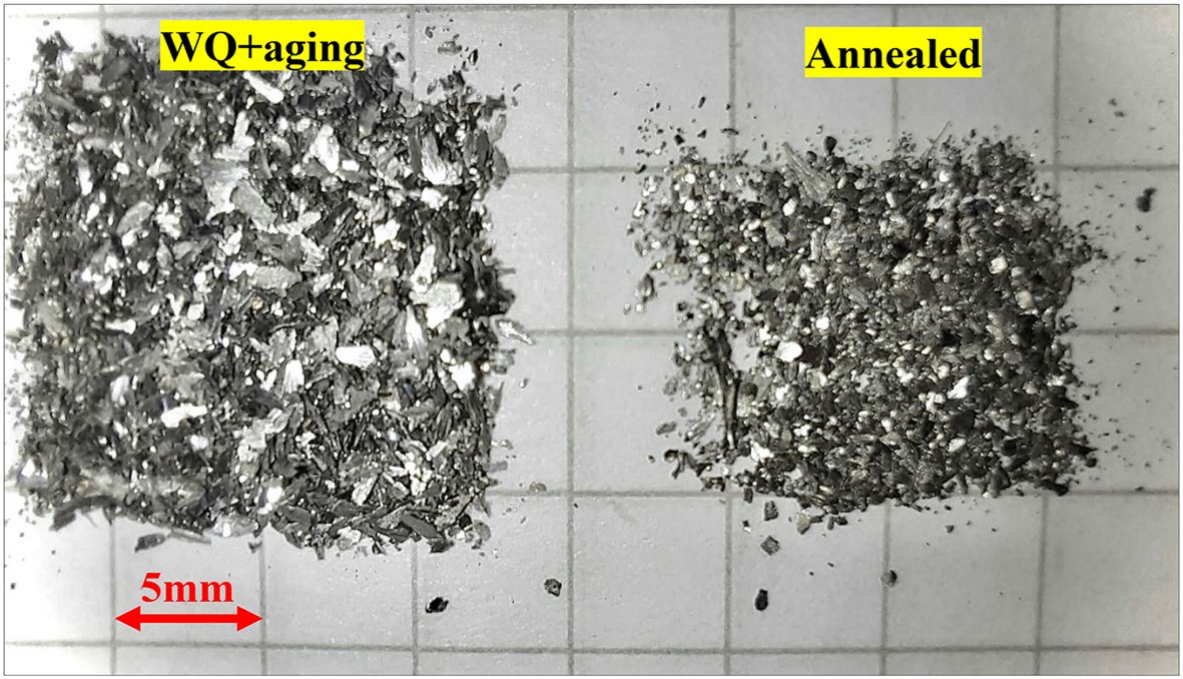
Wear debris of WQ + Aging and annealed specimens at 50 N and 3 m/s.

**Figure 8:**
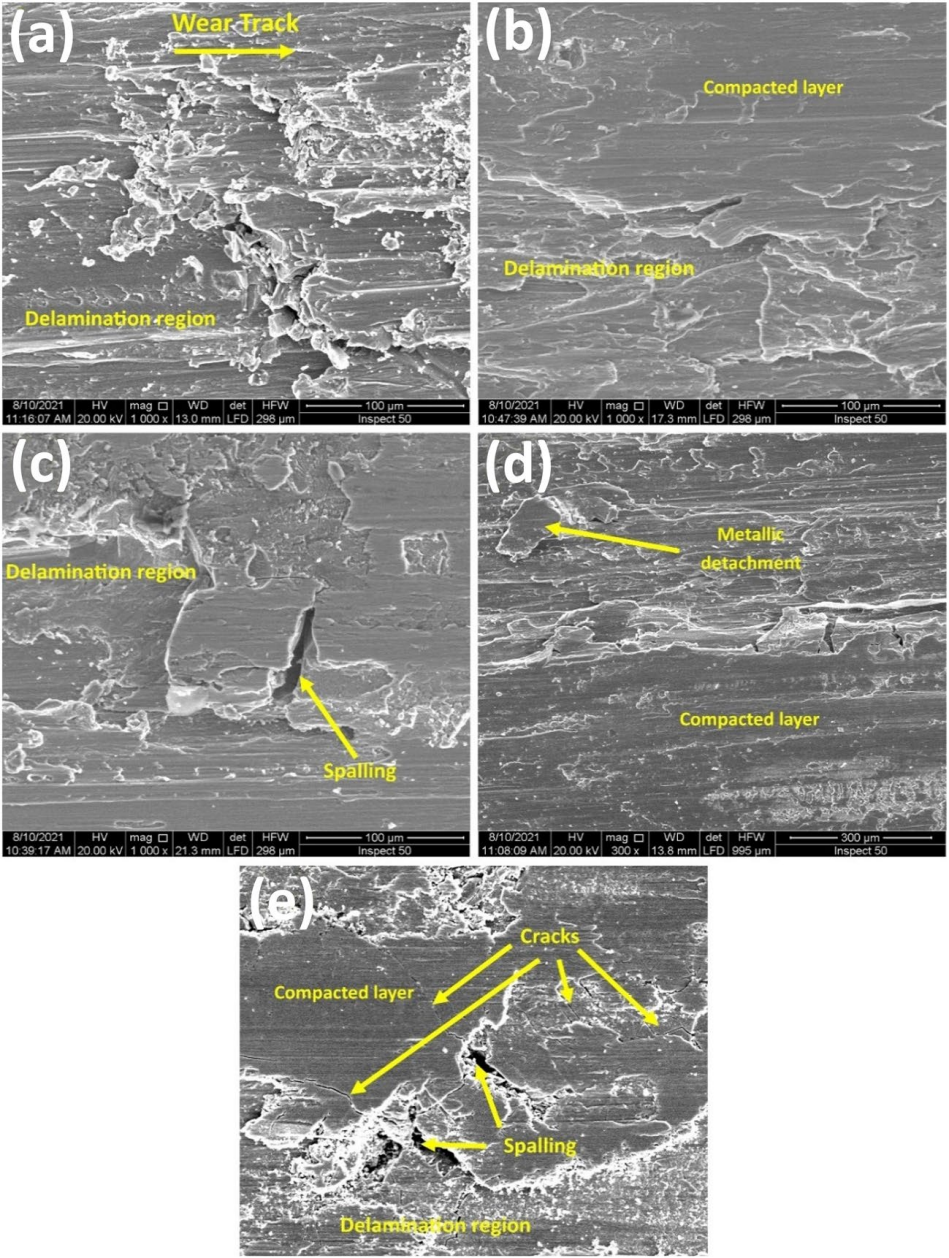
FESEM of worn surfaces under 50 N normal load and 3 m/s sliding speed for (**a**) Annealed, (**b**) AC, (**c**) WQ, (**d**) AC + Aging, and (**e**) WQ + Aging.

Figure 6 reveals an inverse relationship between the surface hardness and wear resistance expressed by WR, where wear rate increase (wear resistance decrease) is associated with hardness increase. By comparison of wear debris collected during testing of the annealed and WQ + Aging samples, it was noticed that the size of debris from WQ + Aging was much larger than that from annealed specimens as shown in Fig. 7. This suggests that the TC21 Ti-alloy undergoes a change in wear behavior from plastic deformation in the annealed condition to more brittle fracture of surfaces in WQ + Aging condition. This suggestion is supported by FESEM results of WQ + Aging worn surface which revealed existing of smoothed compacted layers which are generally damaged in a brittle manner^1^ along with surface spalling and cracks, Fig. 8e. The severe brittleness of the tribolayer of WQ + Aging may be attributed to the presence of a lot of fine platelets of *α*_*s*_ precipitated along grain boundaries which means a lot of voids compared to equiaxed *α* in annealed condition^33^. Those voids can easily link up to form cracks and hence large delamination occurs. Similar results were reported by Sahoo et al.^1,18^ and Feng et al.^34^, they reported an inverse relationship between wear resistance and surface hardness.

**Figure 9:**
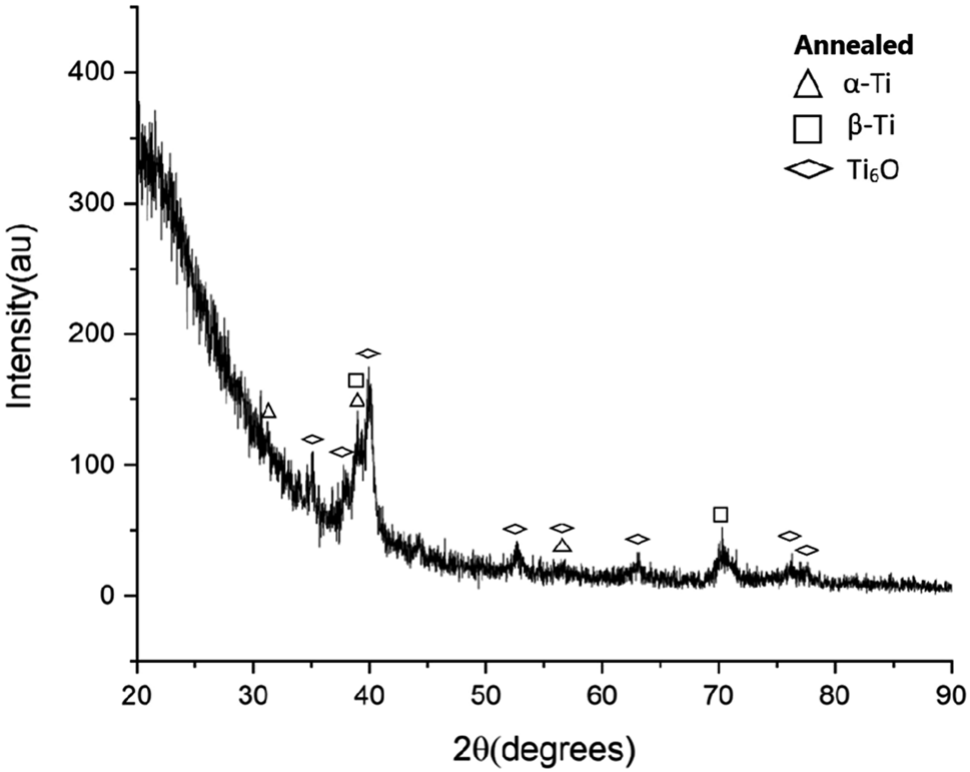
XRD spectrum of the annealed specimen’s debris.

**Figure 10:**
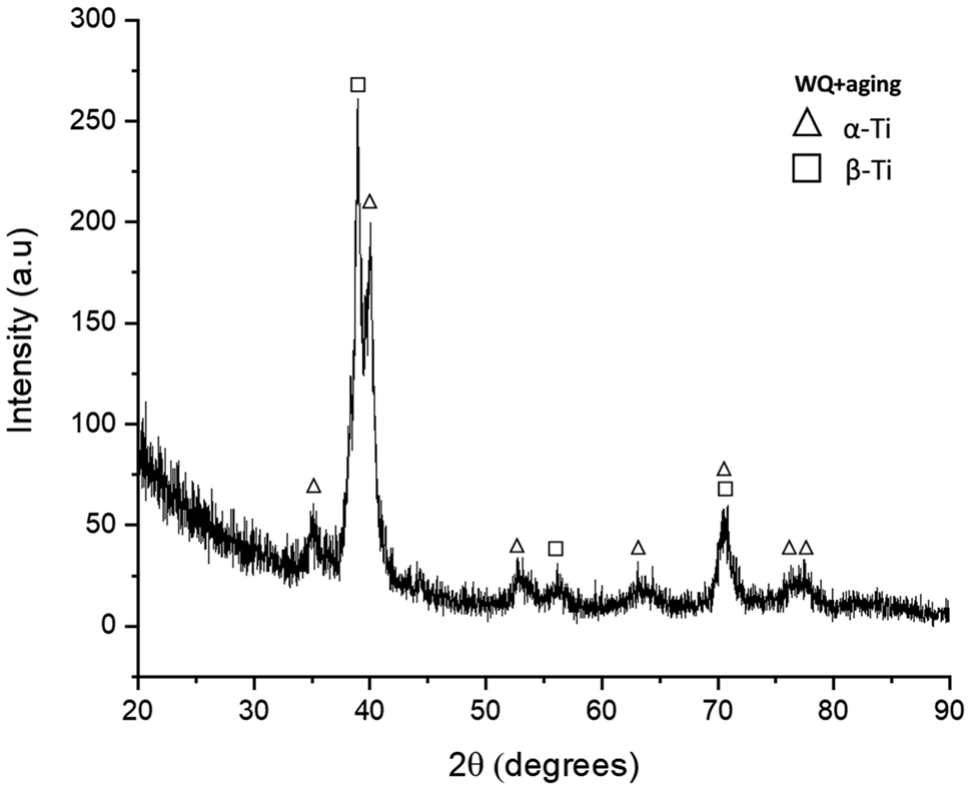
XRD spectrum of the WQ + Aging specimen’s debris.

Furthermore, as the normal load increases, the real area of contact between the two friction mates increases leading to an increase in temperature due to high friction force which is the frictional thermal effect. As a result of low thermal conductivity and high chemical affinity of the titanium especially at high temperatures, a chemical reaction with the ambient oxygen occurred and titanium oxides formed as revealed by the XRD spectrum analysis of wear debris of the annealed specimens as shown in Fig. 9. The presence of the titanium oxide is thought to specimens some protection for the tribolayer of the annealed samples and hence they had a better wear resistance under the extreme conditions of load and speed. On the other hand, the absence of oxides in WQ + aging, Fig. 10 is attributed to a very high rate of removing the tribolayer, and hence no chance for a chemical reaction occurs.

#### Wear mechanisms

**Figure 11:**
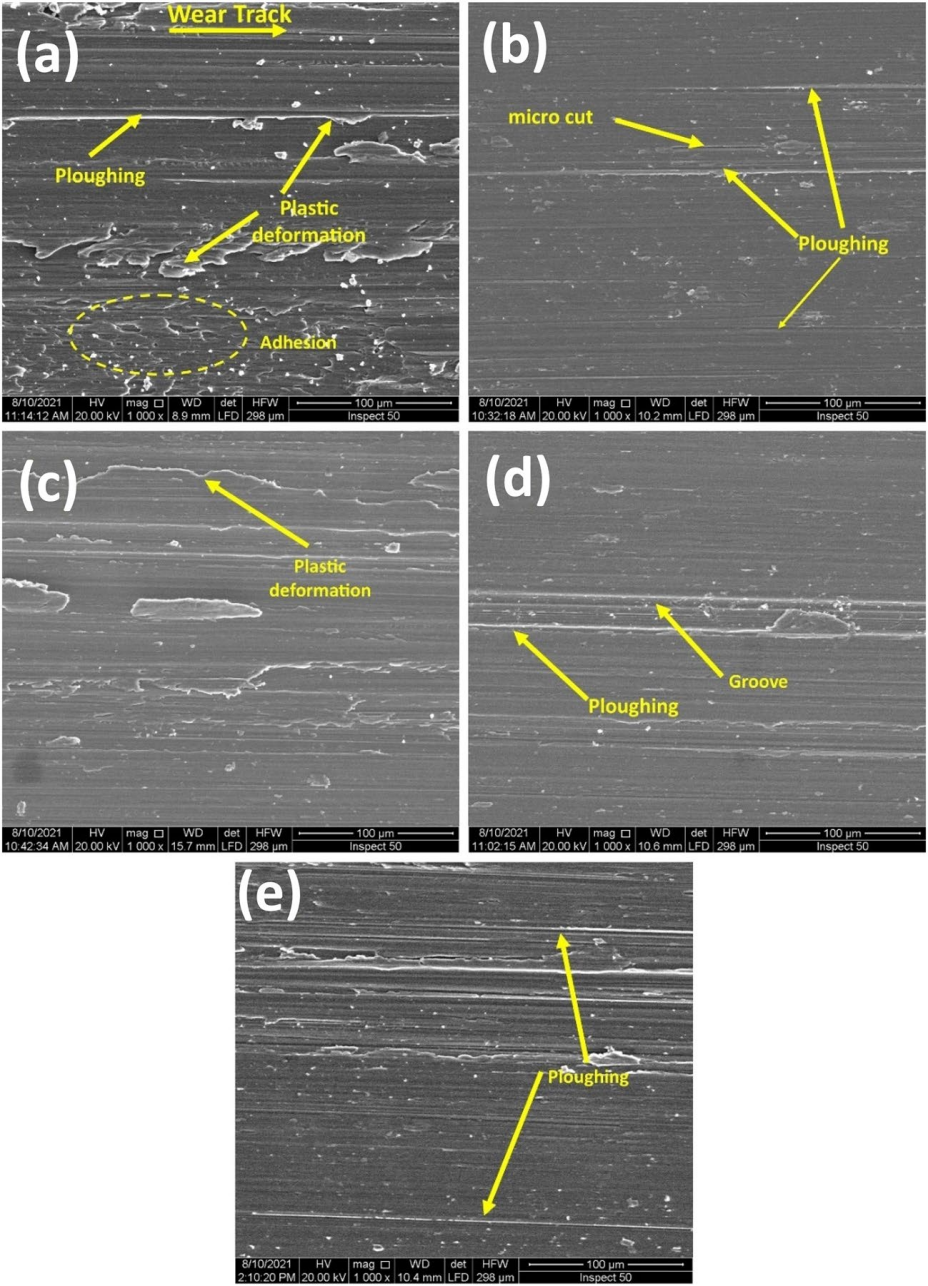
FESEM of worn surfaces under 10 N normal load and 1.5 m/s sliding speed for (**a**) Annealed, (**b**) AC, (**c**) WQ, (**d**) AC + Aging, and (**e**) WQ + Aging.

The morphologies of some worn surfaces obtained under several conditions of load and speed for all different conditions are shown in Figs. 8 and 11. Under low load of 10 N and low speed of 1.5 m/s, the worn surfaces, Fig. 11, showed ploughing remarks caused by debris or asperities on the counter face of the HSS disk with excessive plastic deformation, especially for the annealed condition which also showed some small adhesion marks which may be attributed to its low hardness. Therefore, under those low conditions, the predominant wear mechanism is abrasive wear mechanism. When the testing conditions reach extreme levels i.e., 50 N and 3 m/s, a severe rapture can be observed as a result of delamination and spalling occurring, Fig. 8, due to brittle fracture, especially in WQ + Aging specimen because of its high hardness, Fig. 8d.

### Statistical analysis and modeling

#### Analysis of variance (ANOVA)

**Figure 12:**
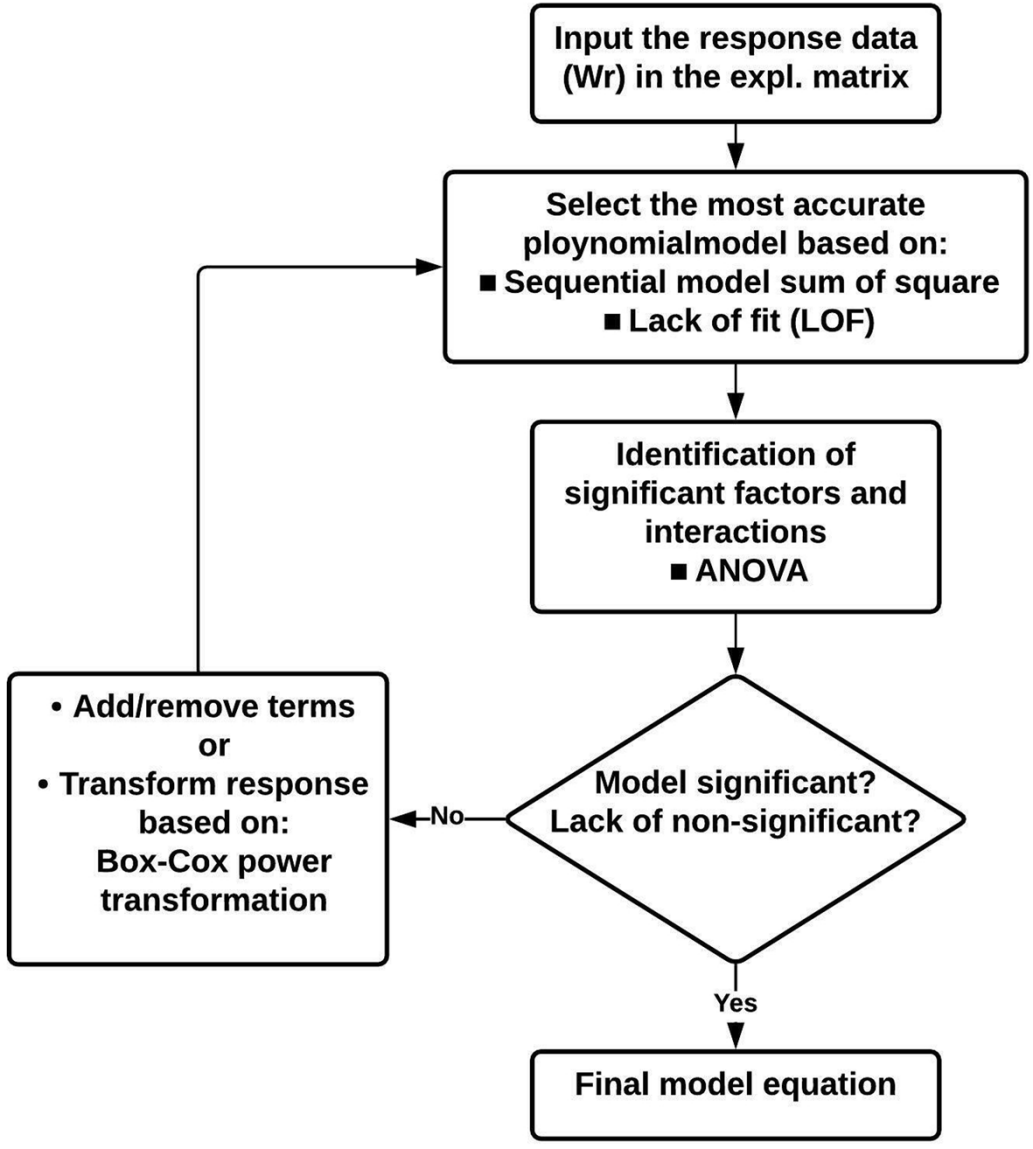
Statistical analysis sequence.

The following chart, Fig. 12, illustrates and summarizes the sequence of statistical analysis used in this study. It involves the analysis of the response variations obtained from the experimental work by a well-established statistical method known as the Analysis of Variance (ANOVA). This was in addition to utilizing the response transformation (Box–Cox power transformation). This transformation is an efficient way to develop an equation for a mathematical model that could have a good fit for the experimental data.

Table 5 shows the ANOVA results of the final improved model based on a 95% confidence level. The results show that the reduced quartic model after transformation is significant (p = 0.0001) with a model F-value of 433.12, which means there is only a 0.01% chance that an F-value this large could occur due to noise. The F-value for lack of fit is 2.05, indicating that it is insignificant (p = 0.1327) when compared to the pure error. Furthermore, all terms with p less than 0.05 are statistically significant. It is obvious that the normal load has been identified as the most significant input factor, followed by the sliding speed and the type of heat treatment. Also, the interaction effect between the load and the speed has been identified as the most significant interaction.

**Table 5:** ANOVA results for reduced quartic model after inverse Sqrt transform.

Source	Sum of squares	df	Mean square	F-value	p-value	
Model	1.43	32	0.0447	433.12	< 0.0001	Significant
A-Load	1.25	1	1.25	12,123.08	< 0.0001	
B-Speed	0.0987	1	0.0987	955.93	< 0.0001	
C-Heat treatment	0.0202	4	0.0050	48.87	< 0.0001	
AB	0.0079	1	0.0079	76.34	< 0.0001	
AC	0.0125	4	0.0031	30.27	< 0.0001	
BC	0.0035	4	0.0009	8.51	0.0003	
A^2^	0.0034	1	0.0034	33.36	< 0.0001	
B^2^	0.0100	1	0.0100	96.42	< 0.0001	
ABC	0.0081	4	0.0020	19.50	< 0.0001	
A^2^B	0.0000	1	0.0000	0.4735	0.4986	
A^2^C	0.0031	4	0.0008	7.61	0.0005	
AB^2^	0.0000	1	0.0000	0.1371	0.7147	
B^2^C	0.0071	4	0.0018	17.08	< 0.0001	
A^2^B^2^	0.0021	1	0.0021	20.39	0.0002	
Residual	0.0023	22	0.0001			
Lack of fit	0.0016	12	0.0001	2.05	0.1327	not significant
Pure error	0.0007	10	0.0001			
Cor total	1.43	54				

**Figure 13:**
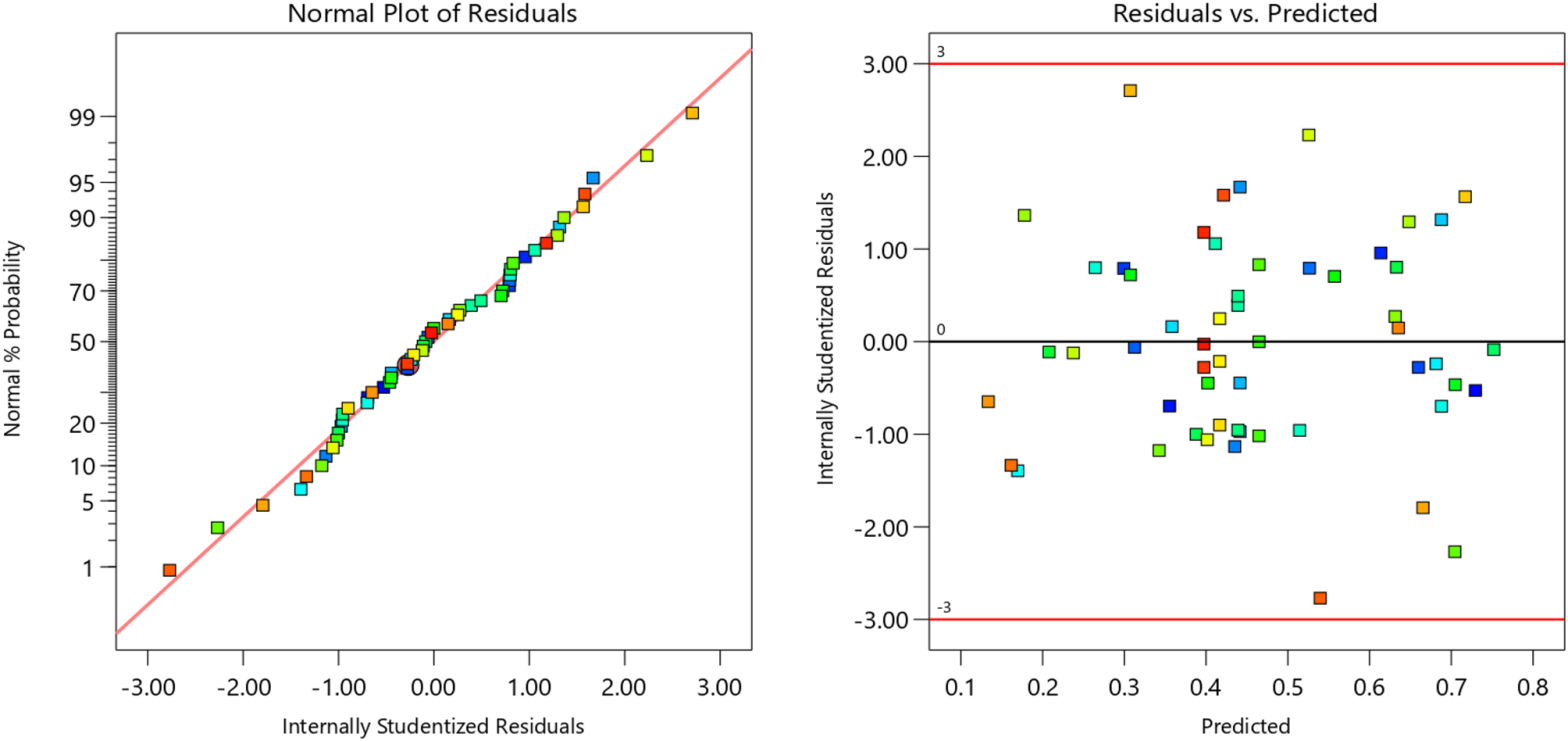
Internally residuals for the final regression model.

The fit statistics, Table 6, show that the coefficient of determination R-squared (R^2^) which is a measure of the amount of variation around the mean that could be explained by the model, i.e., fit-wellness, has a value of 0.9984. That is, the model can explain 99.84% of total variation. In addition, the predicted R^2^ of 0.9813 is so close to the adjusted R^2^ of 0.9961, i.e., the difference is less than 0.2. This indicates the prediction capability of the model is very good. This is supported by the coefficient of variation (C.V), which is the standard deviation expressed as a percentage of the mean, (C.V) = 2.2%, in opposite to C.V = 60.66% before model enhancements. “Adeq Precision” measures the signal-to-noise ratio; 4 is the minimum ratio required. A ratio of 78.56 indicates an adequate signal, and hence, this model can be used to navigate the design space. Figure 13 indicates that the model residuals are normally distributed. Equations from 2 to 6 represent the final empirical equations for different conditions in terms of actual factors.

2 $$ \left({\frac{1}{\sqrt{\text{WR}\times {10}^{3}}}}\right)_{\text{Annealed}}= 0.798859+ 0.019132\times L+0.085580\times S- 0.031117\times L\times S-0.000490\times {L}^{2}-0.037372\times {S}^{2}+0.000525\times {L}^{2}\times S+0.007016\times L\times {S }^{2}-0.000115\times {L}^{2}\times {S }^{2} $$

3 $$ \left({\frac{1}{\sqrt{\text{WR}\times {10}^{3}}}}\right)_{\text{AC}}= 0.444425+ 0.027394\times L+0.321524\times S- 0.035149\times L\times S-0.000508\times {L}^{2}-0.064667\times {S}^{2}+0.000525\times {L}^{2}\times S+0.007016\times L\times {S }^{2}-0.000115\times {L}^{2}\times {S }^{2} $$

4 $$\left({\frac{1}{\sqrt{\text{WR}\times {10}^{3}}}}\right)_{WQ}= 0.593219+ 0.022889\times L+0.298113\times S- 0.033129\times L\times S-0.000498\times {L}^{2}-0.080619\times {S}^{2}+0.000525\times {L}^{2}\times S+0.007016\times L\times {S }^{2}-0.000115\times {L}^{2}\times {S }^{2} $$

5 $$\left({\frac{1}{\sqrt{\text{WR}\times {10}^{3}}}}\right)_{\text{AC}+\text{aging}}=0.728070+0.025967\times L+0.078446\times S- 0.033640\times L\times S-0.000536\times {L}^{2}-0.023861\times {S}^{2}+0.000525\times {L}^{2}\times S+0.007016\times L\times {S }^{2}-0.000115\times {L}^{2}\times {S }^{2} $$

6 $$\left({\frac{1}{\sqrt{\text{WR}\times {10}^{3}}}}\right)_{\text{WQ}+\text{aging}}= 0.982825+0.029305\times L+0.192730\times S- 0.034146\times L\times S - 0.000599\times {L}^{2}-0.040683\times {S}^{2}+0.000525\times {L}^{2}\times S+0.007016\times L\times {S }^{2}-0.000115\times {L}^{2}\times {S }^{2} $$

where, L = load in (N) and S = sliding speed (m/s).

**Table 6:** Fit statistics for reduced quartic model after inverse Sqrt transform.

Std. dev.	0.0102	R^2^	0.9984
Mean	0.4616	Adjusted R^2^	0.9961
C.V. %	2.20	Predicted R^2^	0.9813
		Adeq precision	78.5628

####  Model graphs

**Figure 14:**
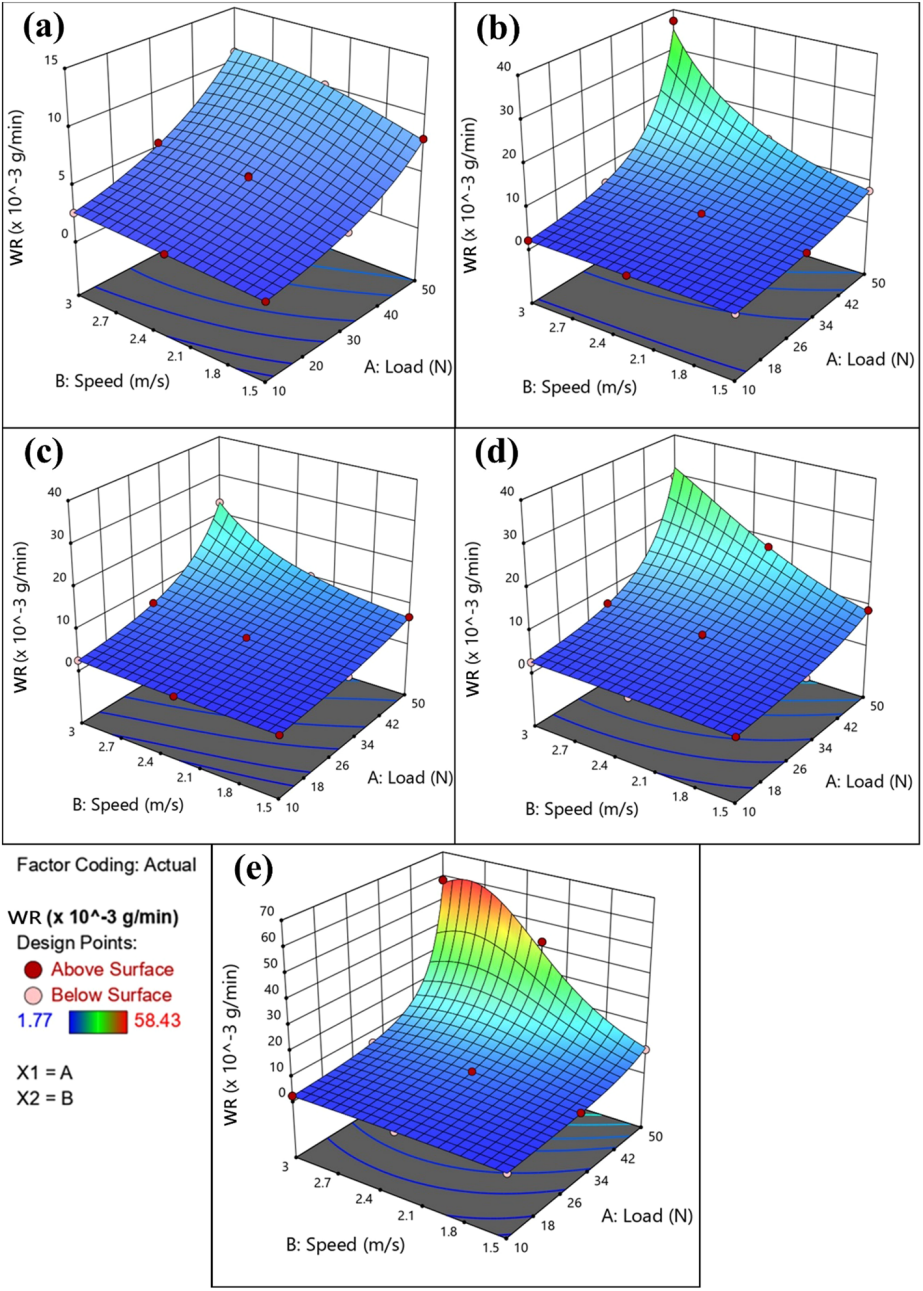
3D response surface for WR of (**a**) annealed, (**b**) AC, (**c**) WQ, (**d**) AC + Aging, and (**e**) WQ + Aging.

**Figure 15:**
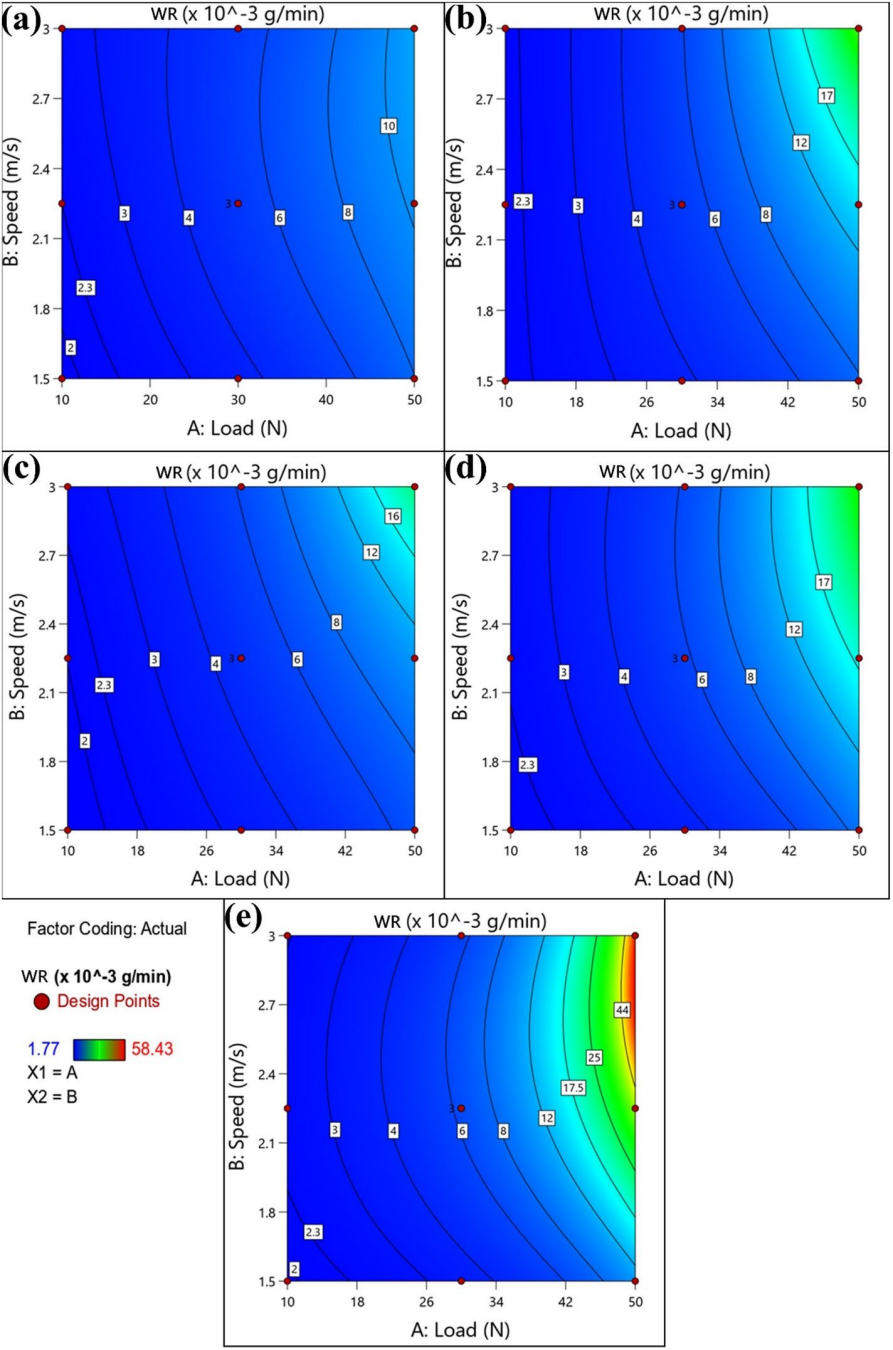
2D contour plots for WR of (**a**) annealed, (**b**) AC, (**c**) WQ, (**d**) AC + Aging, and (**e**) WQ + Aging.

To illustrate the combined effect of independent parameters on the response (WR), 3D response surface plots and 2D contour plots are constructed for all heat treatment conditions, as shown in Figs. 14 and 15, respectively. According to those plots, the wear rate increases with the increase in normal load and sliding speed, especially at high levels. In addition, this increase in wear rate is most dramatic in the WQ + Aging condition, Fig. 14e, while it is too small in the annealed condition, Fig. 14a.

#### Model validation

To validate the obtained regression model, confirmation tests were carried out. The input parameters chosen within the design space constraints. Table 7 summarizes the input parameters levels applied, the corresponding experimental WR, and predicted WR. From the results, the model has a good prediction ability with average absolute error equal to 3.91%. In addition, all predicted values are within the 95% prediction interval (PI) limits of the model.

**Table 7:** Comparison between experimental and predicted wear rate.

No.	Load (N)	Speed (m/s)	HT	Expt. WR (g/min) × 10^–3^	Predict. WR (g/min) × 10^–3^	95% PI low	95% PI High	% of abs. error
1	20	2.5	Annealed	3.47	3.62	3.31	3.97	4.32
2	30	2.75	Annealed	5.54	5.45	4.88	6.11	1.62
3	40	2	Annealed	6.79	6.86	6.08	7.78	1.03
4	20	2.5	AC	3.23	3.35	3.07	3.65	3.72
5	30	2.75	AC	5.42	5.81	5.18	6.53	7.2
6	40	2	AC	7.11	7.36	6.49	8.39	3.52
7	20	2.5	WQ	3.49	3.28	3.02	3.59	6.02
8	30	2.75	WQ	5.53	5.69	5.07	6.39	2.89
9	40	2	WQ	5.97	6.11	5.45	6.88	2.35
10	20	2.5	AC + aging	3.68	3.79	3.46	4.16	2.99
11	30	2.75	AC + aging	5.94	6.39	5.66	7.23	7.58
12	40	2	AC + aging	7.81	8.21	7.18	9.42	5.12
13	20	2.5	WQ + aging	3.97	3.85	3.51	4.22	3.02
14	30	2.75	WQ + aging	6.19	6.42	5.7	7.27	3.72
15	40	2	WQ + aging	9.93	10.28	8.86	11.99	3.52
Average								3.91

#### Optimization

**Figure 16:**
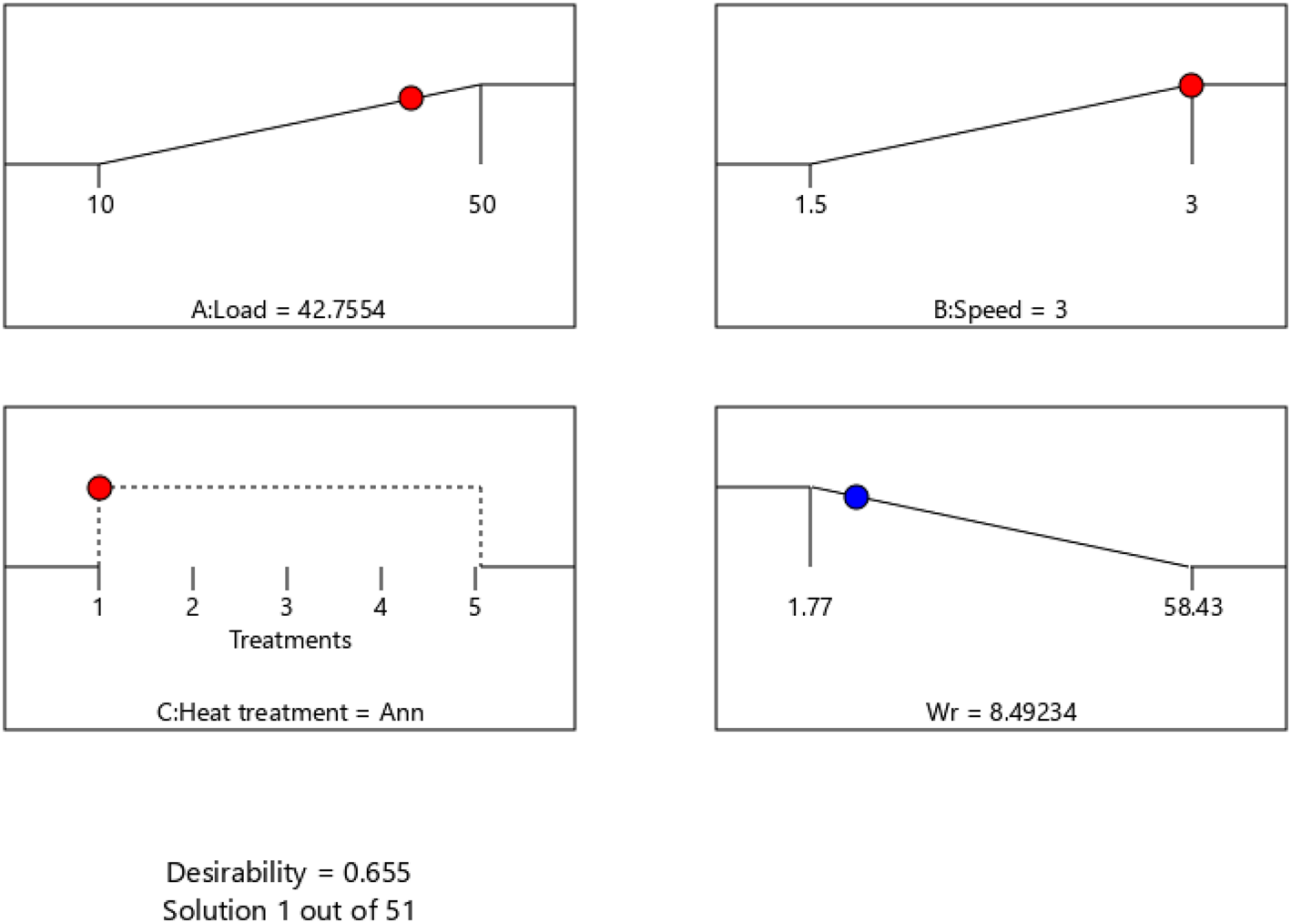
Optimum set of input parameters necessary to get the minimum WR under extreme conditions.

The best treatment is considered that one giving a microstructure withstand extreme operating conditions i.e., maximum normal load and maximum sliding speed but shows the minimum wear rate. According to this optimization criteria and by using the regression model equations, the optimum solution is shown in Fig. 16. The optimum set of input parameters are 42.75 N normal load, 3 m/s sliding speed and annealed condition (Equiaxed microstructure) which give optimum wear rate of 8.49 g/min with maximum desirability of 0.655 which means the gool of optimization is achieved by 65.5%. Table 8 summarizes confirmation test results at optimum conditions, the experimental WR within the 95% prediction interval (PI) limits of the model with average absolute error of 6.04%.

**Table 8:** Confirmation test results at optimum conditions.

No.	Load (N)	Speed (m/s)	HT	Expt. WR (g/min) × 10^−3^	Predicted WR (g/min) × 10^−3^	95% PI low	95% PI high	% Absolute error
1	42.75	3	Ann	8.93	8.49	7.31	9.93	4.93
2	42.75	3	Ann	9.15	8.49	7.31	9.93	7.21
3	42.75	3	Ann	9.03	8.49	7.31	9.93	5.98
Average								6.04

## Conclusions

- With respect to annealed specimens (38 HRC), the minimum hardness achieved by WQ specimens of 36 HRC, while the maximum hardness achieved by WQ + Aging specimens of 49 HRC.
- Under extreme wear condition (50 N, 3 m/s), although WQ + aging specimens had the maximum hardness, they showed the worst wear resistance. While, the annealed ones showed the best wear resistance regardless having much lower hardness.
- Abrasive wear mechanism is predominant under low wear conditions (10 N, 1.5 m/s) while delamination wear mechanism is predominant under extreme conditions.
- Using RSM, regression model for wear resistance expressed in wear rate has been developed as a function in normal load, sliding and type of heat treatment. Based on ANOVA, the normal load has been identified as the most significant input factor followed by the sliding speed and the type of heat treatment. Also, the interaction effect between the load and the speed has been identified as the most significant interaction.
- Model validation results revealed the experimental results are within 95% prediction interval of the model with average absolute error of 3.91% hence, the developed model is valid to predict WR within the design space.
- The obtained model was used to predict the optimum levels of input factors required to get the minimum wear rate under severe conditions of load and speed. Experimental results showed that the actual WR under those optimum levels is close to the predicted one with average absolute error of 6.04%.

## Data Availability

All data generated or analyzed during this study are included in this published article.
